# Proposal for a Paradigm Shift in Personalized Medicine for Patients with a Maxillary Edentulous Jaw by ENT Specialist and Dentist Cooperation

**DOI:** 10.3390/jpm12081289

**Published:** 2022-08-05

**Authors:** Yuh Baba, Yasumasa Kato, Keiso Takahashi

**Affiliations:** 1 Department of General Clinical Medicine, Ohu University School of Dentistry, 31-1, Mitsumido, Tomita-machi, Koriyama City 963-8611, Fukushima, Japan; 2 Department of Oral Function and Molecular Biology, Ohu University School of Dentistry, 31-1, Mitsumido, Tomita-machi, Koriyama City 963-8611, Fukushima, Japan; 3 Division of Periodontics, Department of Conservative Dentistry, Ohu University School of Dentistry, 31-1, Mitsumido, Tomita-machi, Koriyama City 963-8611, Fukushima, Japan

**Keywords:** oral implant, maxillary sinusitis, sinus lift, paradigm shift in personalized medicine, maxillary sinus pathologies, anatomic variations, ENT and dentist cooperation

## Abstract

With the spread of oral implant therapy, serious medical complications related to implant surgery are becoming a social problem. Although the number of complications after implant surgery in the edentulous jaw is decreasing in Japan, maxillary-sinus-related complications (MSRCs) have reached the highest number since 2012. It is essential to identify and eliminate possible predisposing risk factors for MSRCs at an early stage to prevent MSRCs. In this review article, we highlight the causal factors of postoperative complications with or without sinus augmentation for the maxillary molar region to achieve optimal treatment outcomes and reduce complications. In particular, we focus on anatomical variations that can cause the impairment of maxillary sinus drainage. Furthermore, we emphasize that the paradigm for personalized medicine for patients with a maxillary edentulous jaw by ENT specialist and dentist cooperation is shifting from the traditional assessment of maxillary sinus pathologies alone to the new assessment of anatomic variations that can cause the impairment of maxillary sinus drainage in addition to maxillary sinus pathologies.

## 1. Introduction

**Figure 1:**
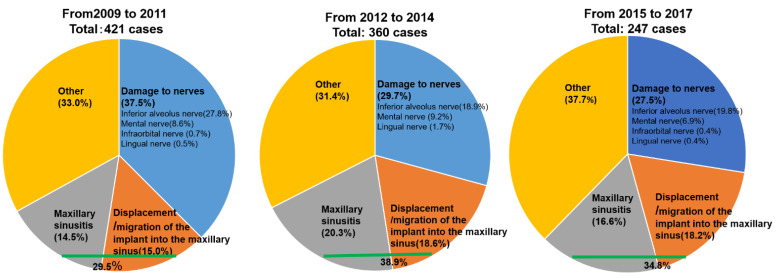
Before 2012, the rate of damage to nerves (37.5%) was higher than that of MSRCs (29.5%) (both maxillary sinusitis and displacement or migration of the implant into the maxillary sinus). In contrast, since 2012, the rate of MSRCs has been higher than that of damage to nerves due to complications after implant surgery in Japan. Adapted from References [5,6,7].

Although the use of oral implants in the oral rehabilitation of edentulous patients is widely accepted, serious medical complications related to implant surgery are a social problem [1]. The serious complications include intra- or postoperative bleeding, hematoma formation, varying degrees of neurosensory alteration, maxillary sinusitis, displacement/migration of the implant into the maxillary sinus, and so on [2,3,4]. Although the number of complications after implant surgery in the edentulous jaw is decreasing in Japan, maxillary-sinus-related complications (MSRCs) (both maxillary sinusitis and displacement or migration of the implant into the maxillary sinus) have remained the highest after implant surgery since 2012 (Figure 1) [5,6,7]. The approach for the latter is thought to be relatively easy when the surgeon uses functional endoscopic sinus surgery (FESS) and/or an oral approach (OA) technique [4]. Therefore, ear, nose, and throat (ENT) specialists and dentists should take particular care to prevent maxillary sinusitis after implant surgery.

**Figure 2:**
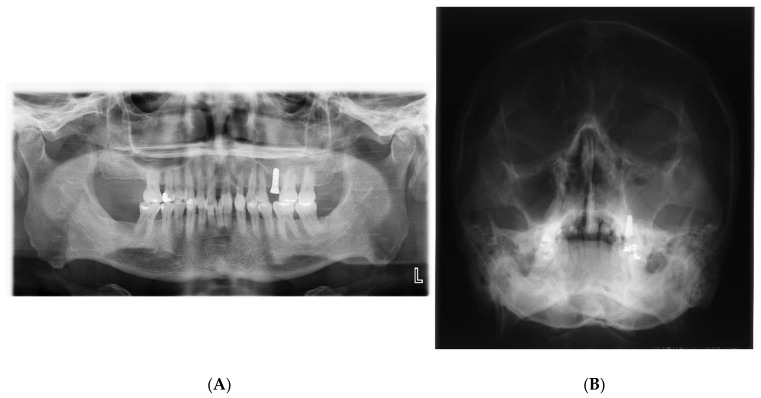
(**A**) During implant placement, the surgeon pierced the maxillary sinus mucosa, and the postoperative panoramic photograph shows that the implant body slightly protrudes into the left maxillary sinus floor; (**B**) 7 days after the operation, the upper left maxillary sinus was opaque, suggesting acute left maxillary sinusitis. (unpublished data).

The possible causes of chronic maxillary sinusitis after dental implantation caused by surgeons include sinus penetration by the implant (Figure 2), perforation of the Schneiderian membrane during the sinus lift, and so on [3]. However, maxillary sinusitis rarely occurs after sinus penetration by implant or perforation of the Schneiderian membrane during a sinus lift, because the implant body or bone graft is sterilized and clean [8]. Therefore, it is not clear whether sinus penetration by the implant or perforation of the Schneiderian membrane during a sinus lift is a possible factor predisposing the patient to maxillary sinusitis after dental implantation.

In order to prevent this complication, we need to identify possible causal factors prior to implant surgery and eliminate these factors before surgery. In this study, we highlight the main possible causal and risk factors for postoperative complications after implant surgery with or without sinus augmentation of the maxilla molar region to achieve optimal treatment outcomes and reduce complications. In particular, we need to keep in mind anatomical variations, which can cause the impairment of maxillary sinus drainage, in addition to maxillary sinus pathologies. Furthermore, we propose a new paradigm for personalized medicine for patients with a maxillary edentulous jaw through ENT and dentist cooperation.

## 2. Identification of the Possible Causal and Risk Factors for Maxillary Sinusitis after Implant Surgery with or without Sinus Augmentation

### 2.1. Untreated or Incompletely Managed Diabetes Mellitus and Smoking

Dentists should ask patients who will be undergoing implant surgery about any untreated or incompletely managed diabetes mellitus (DM) or smoking history. Although it is unclear whether poorly managed DM can facilitate maxillary sinusitis after implant surgery, a systematic review suggested that DM is associated with a greater risk of peri-implantitis, independent of smoking [9]. Therefore, we think that poorly managed DM and smoking may exaggerate postoperative maxillary sinusitis through the pathway of the implant body with the bacterial biofilm attached to the maxillary sinus.

### 2.2. Dental Causes such as Poor Oral Hygiene, Persistent/Recurrent Periodontitis, and Peri-Implantitis

**Figure 3:**
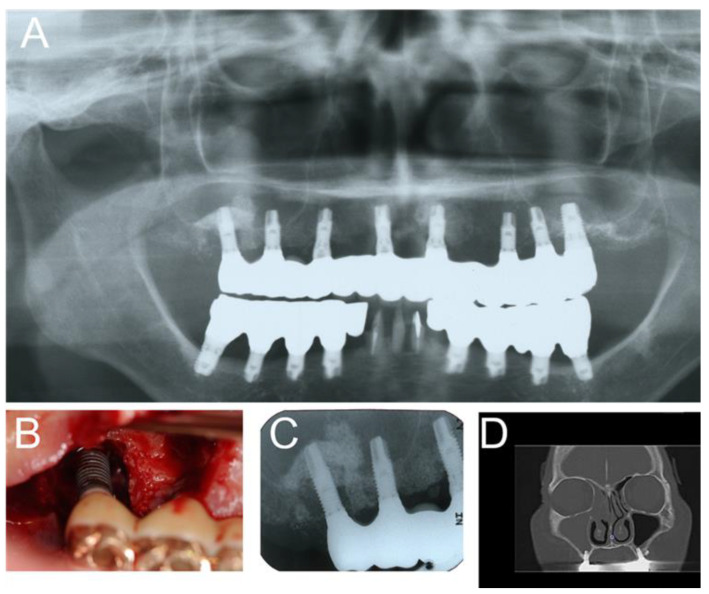
Severe peri-implantitis in the upper right molar region was observed 10 years after implant therapy with sinus augmentation. (**A**) Panoramic radiography; (**B**) oral photograph; (**C**) intraoral radiograph; (**D**) paranasal CT shows right rhinosinusitis. In this case, chronic maxillary sinusitis occurred through the pathway from peri-implantitis to the maxillary sinus for a long period. Therefore, FESS was performed (unpublished data).

Severe peri-implantitis may cause chronic maxillary sinusitis (Figure 3). The dental causes of peri-implantitis include poor oral hygiene, persistent/recurrent periodontitis, and so on [10]. Therefore, we think that peri-implantitis can be a possible causal/risk factor for postoperative complications after implant surgery.

### 2.3. Alterations of the Anatomical Structure That Can Facilitate the Impairment of the Maxillary Sinus Drainage Pathway

Because maxillary secretions are drained into the middle meatus through the natural ostium of the maxillary sinus, aberrant anatomy that affects the impairment (stenosis) of the maxillary sinus drainage pathway may be a predisposing factor for maxillary sinusitis after maxillary implantation.

#### 2.3.1. Deviated Nasal Septum

**Figure 4:**
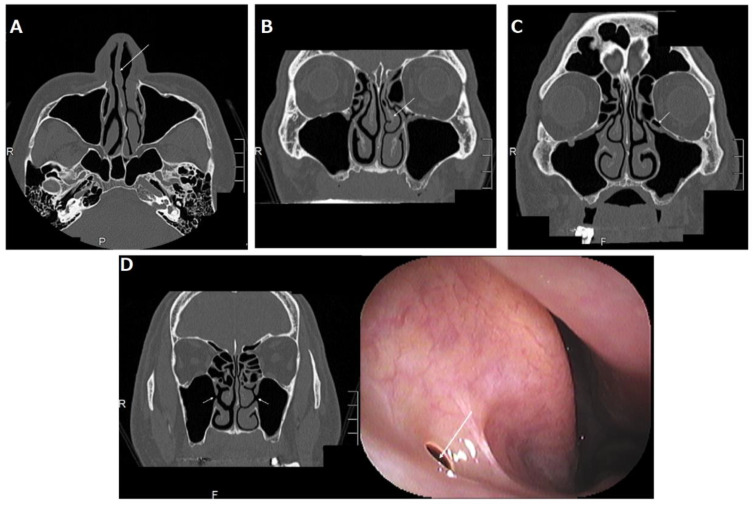
(**A**) Right nasal septal deviation (arrow); (**B**) left paradoxical middle turbinate (arrow) and left concha bullosa of the middle turbinate; (**C**) left Haller cell (arrow); (**D**) bilateral accessory ostium of the maxillary sinus (arrow in CT (left panel)) and right accessory ostium (arrow in nasal endoscopic examination (right panel)). This was originally published in *Case Reports in Otolaryngology*: Suzuki-Yamazaki, M.; Takahashi, K.; Takada, S.; Kato, Y.; Baba, Y. “A successful treatment regimen for the prevention of sinusitis after maxillary sinus floor elevation surgery in a high-risk case.” *Case Rep. Otolaryngol*. 2020, 2020, 6869805 [16].

A systematic review indicated that a deviated nasal septum is associated with an increased prevalence of rhinosinusitis [11] because the curvature of the convexness of the nasal septum laterally displaces the middle turbinate and uncinated process, narrowing the ethmoidal infundibulum and reducing the ventilation and drainage of the maxillary sinus (Figure 4A).

#### 2.3.2. Concha Bullosa or Paradoxical Middle Turbinate

Fadda et al. showed that anatomical variations in the middle turbinate, such as the concha bullosa and paradoxical middle turbinate, are associated with an increased prevalence of rhinosinusitis [12] because they laterally displace the uncinated process, narrowing the ethmoidal infundibulum and reducing ventilation and drainage of the maxillary sinus (Figure 4B).

#### 2.3.3. Haller Cells

Haller cells are defined as infraorbital ethmoid cells. Haller cells also cause rhinosinusitis [12] because the presence of Haller cells can induce the stenosis of the ethmoidal infundibulum, consequently reducing the ventilation and drainage of the maxillary sinus (Figure 4C).

#### 2.3.4. Accessory Ostium

The accessory ostium is located behind the maxillary natural ostium. Doctors should be careful not to confuse it with a natural ostium on computed tomography (CT) (left panel in Figure 4D). The natural ostium cannot be confirmed by nasal endoscopy, but the accessory ostium can be confirmed by endoscopy (right panel of Figure 4D). Although some doctors think that the accessory ostium might improve drainage of the maxillary sinus, it may pose a risk for maxillary sinusitis, because the presence of an accessory ostium can lead to chronic maxillary sinusitis by recirculation of mucus secretions, as well as a decrease in the drainage function of the maxillary sinus [13].

Lee et al. reported a higher rate of complications in patients with anatomic variants such as Haller cells, deviated nasal septum, concha bullosa, and paradoxical curvature; among these risk factors, Haller cells showed a statistically significant association with postoperative maxillary sinusitis after sinus lifting [14]. Furthermore, another study also showed that large Haller cells can be predisposing factors for developing maxillary sinusitis after a sinus lift [15].

## 3. Common Maxillary Sinus Pathologies before Maxillary Implantation

**Figure 5:**
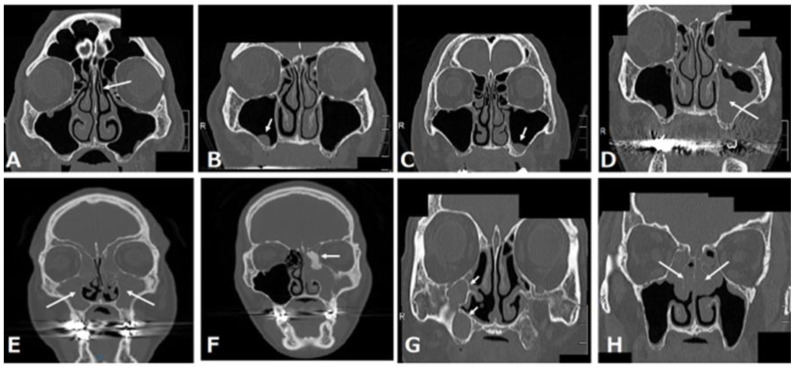
(**A**) Almost normal maxillary sinus and left concha bullosa of the middle turbinate (arrow); (**B**) retention cyst or solitary polyp in the right maxillary sinus (arrow); (**C**) mucosal thickening in the left maxillary sinus (arrow); (**D**) air-fluid level or fluid accumulation in the left maxillary sinus (most often indicating acute rhinosinusitis) (arrow); (**E**) total opacification of the maxillary sinus or other paranasal sinuses (most often indicating chronic rhinosinusitis) (arrow); (**F**) left chronic sinusitis with ethmoidal osteoma (arrow); (**G**) right two postoperative maxillary cysts (POMC) (arrow); (**H**) eosinophilic chronic rhinosinusitis (arrow).

The common maxillary sinus pathologies that can be identified on coronal CT images of the paranasal sinus at our institution are shown in Figure 5. These pathologies are classified into three pathologies: those that need treatment, those that do not need treatment, and those that are not candidates for maxillary implantation. A normal maxillary sinus, retention cyst or solitary polyp, and mucosal thickening in the maxillary sinus do not need treatment before maxillary implantation (Figure 5A–C), whereas preoperative sinusitis needs treatment because it is a cause of the development of postoperative maxillary sinusitis (Figure 5D–F) [17]. Patients with a postoperative maxillary cyst also need treatment (Figure 5G). Patients with eosinophilic chronic rhinosinusitis are not suitable for maxillary implantation (Figure 5H).

### 3.1. Retention Cyst or Solitary Polyp

A retention cyst is described as one of the most common pathological findings of the maxillary sinus and usually presents as a dome-shaped radiopaque soft-tissue mass attached to the bony walls of the sinus (Figure 5B). Retention cysts are benign lesions that can originate from the accumulation of fluids inside the sinus membrane, which result from a ductal obstruction of the seromucous glands [18]. When patients with a small retention cyst or polyp have no complaints, ENT treatments are not required. Furthermore, the placement of dental implants after a sinus lift in patients with retention cysts is safe and presents a high chance of survival, regardless of whether the lesion is removed [19]. Therefore, we think that patients with a small cyst or solitary polyp in the maxillary sinus can still receive a maxillary implant with or without a sinus lift.

### 3.2. Mucosal Thickening in the Maxillary Sinus

Patients with mucosal thickening that is less than one-third to one-half of the maxillary sinus height and localized to the lesion around the teeth (Figure 5C) can still receive a maxillary implant with or without a sinus lift [20,21].

### 3.3. Acute Rhinosinusitis

Rhinosinusitis is defined as inflammation of one or more paranasal sinuses; acute rhinosinusitis is defined by symptoms such as nasal congestion, rhinorrhea, facial pain, hyposmia, and sneezing, which last less than 12 weeks [22]. Imaging often indicates air-fluid levels or fluid accumulation (Figure 5D). After the possibility of coronavirus disease 2019 (COVID-19) is ruled out by a severe acute respiratory syndrome coronavirus 2 (SARS-CoV-2) RT-PCR test—because the clinical manifestations of rhinosinusitis are similar to the symptoms of COVID-19 [23] and patients recover from the illness by conservative ENT treatment—they can still receive a maxillary implant with or without a sinus lift.

### 3.4. Chronic Rhinosinusitis

Chronic rhinosinusitis is defined as symptoms that last more than 12 weeks [22]. Chronic rhinosinusitis is classified into two categories: neutrophilic and eosinophilic. Furthermore, neutrophilic chronic sinusitis has two patterns: with or without a benign tumor.

#### 3.4.1. Neutrophilic Chronic Rhinosinusitis

CT often shows almost all opacification of the maxillary sinus or other paranasal sinuses (Figure 5E). After conservative and/or surgical treatment by FESS, patients can still receive an implant treatment with or without a sinus lift [24].

#### 3.4.2. Neutrophilic Chronic Rhinosinusitis with a Benign Tumor Such as Osteoma

Neutrophilic chronic rhinosinusitis with a benign tumor in the paranasal sinuses is a subtype of neutrophilic chronic sinusitis (Figure 5F). This disease can be considered to result from the obstruction of the natural ostium of the maxillary sinus by the tumor. The surgical options for this disease are an endoscopic procedure or a combined external and endoscopic procedure [25]. If a surgeon selects the former and can achieve complete resection, patients can still receive a maxillary implant with or without a sinus lift. Otherwise, when a surgeon selects the combined approach, dentists should consider the possibility of a postoperative maxillary cyst (POMC) at the time of the maxillary implant.

#### 3.4.3. Eosinophilic Chronic Rhinosinusitis (ECRS)

ECRS is recognized as refractory chronic rhinosinusitis despite the combination of macrolide therapy and FESS, which is effective for neutrophilic chronic rhinosinusitis [26]. ECRS has a strong tendency for recurrence after FESS. CT scan images of ECRS patients show them to be ethmoid-sinus-dominant (Figure 5H), while CT of neutrophilic chronic sinusitis shows maxillary predominance in the early stages. Patients with ECRS tend to be more likely to develop rhinosinusitis symptoms after maxillary implant treatment, so they are not suitable for implant treatment or other prostheses such as dentures; implant-supported overdentures are recommended as an alternative method to avoid maxillary implant treatment [27].

### 3.5. POMC

POMC is a delayed complication of maxillary sinus surgery, such as Caldwell–Luc surgery, which is a radical technique used to remove infection and diseased mucosa from the maxillary sinus (Figure 5G). Marsupialization with drainage of a POMC should be performed before the insertion of an implant, in order to prevent implant failure resulting from possible bone destruction around the dental implant following the expansion of the POMC [28].

## 4. Discussion

**Figure 6:**
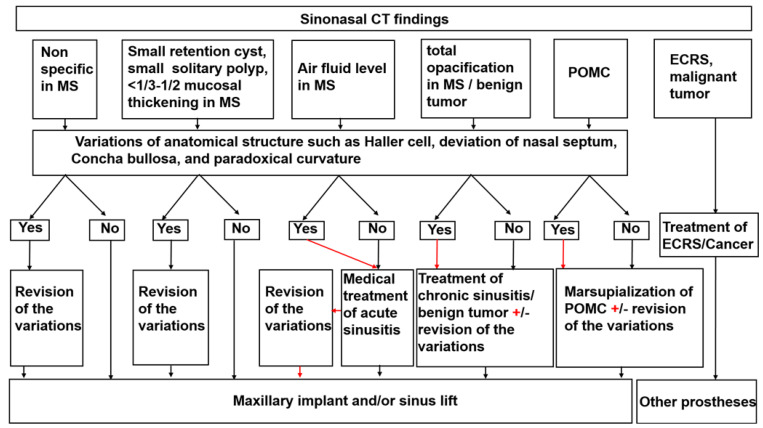
Evaluation and management protocol based on sinonasal CT findings.

As shown in Section 3 [20,21,29], previous personalized medicine for patients with a maxillary edentulous jaw by ENT specialist and dentist cooperation was selected according to the maxillary sinus pathologies. Some clinicians pointed out that some anatomic variants such as Haller cells, a deviated nasal septum, concha bullosa, and paradoxical curvature can be risk factors of maxillary sinusitis after maxillary implantation, even if the maxillary sinus is normal before the maxillary implantation [14,15]. Therefore, when these anatomical risk factors are discovered before maxillary implantation, conservative or surgical prevention is recommended [30]. However, when personalized medicine is chosen according to maxillary sinus pathologies, we have serious concerns about the possibility of overlooking high-risk patients with such anatomic variants whose maxillary sinus is normal, as shown in Figure 4 and Figure 5A. Rhinosinusitis tends to result from any anatomic variations that can cause stenosis of maxillary drainage pathways [11,12,13]. Therefore, personalized medicine for patients with a maxillary edentulous jaw by ENT and dentist cooperation should be chosen according to both anatomical variations and maxillary sinus pathologies. Here, we propose an evaluation and management protocol based on sinonasal CT findings (Figure 6). When there is a normal lesion, small retention cyst, small solitary polyp, or mucosal thickening that is less than one-third to one-half of the maxillary sinus height in the maxillary sinus, the patients can receive a maxillary implant with or without revision of the anatomical variations. Patients with acute and/or chronic rhinosinusitis, benign tumor, or POMC can receive a maxillary implant with or without revision of the anatomical variations after recovery from these illnesses. Patients with refractory disorders such as ECRS and malignant tumors are not suitable for maxillary implantation with sinus elevation; as an alternative, other prostheses such as dentures, implant-supported overdentures, and the sinus slot technique may be candidates as an alternative method [31]. Under this new paradigm, there is no patient with chronic rhinosinusitis who might need FESS after maxillary implantation at our institution (unpublished data).

This study has some limitations. The first limitation is the small number of participants under this new paradigm at our institution. The number is less than the eighty-four that was previously reported by Chen et al. [21]. We think the reason for this is that the number of patients planning to attend the dental clinic in order to receive maxillary implantation has decreased during the COVID-19 pandemic. The second limitation is deficient comparison with the traditional paradigm. Therefore, we are conducting further research into whether the clinical outcome under this new proposal is superior to that under the traditional paradigm by increasing the number of participants under this new paradigm.

Before the COVID-19 pandemic, ENT specialists tended to use both nasal endoscopy and sinonasal CT for these anatomic variations [12,21,30]. However, during the COVID-19 pandemic, it was recommended that nasal endoscopy not be used except in emergencies, because a nasal endoscopy examination may carry the risk of possible droplet infection [32]. Therefore, we recommend that ENT specialists evaluate both anatomic variations and maxillary pathologies using paranasal CT during the COVID-19 pandemic because paranasal CT findings correspond to those of a nasal endoscopy [13]. Promising alternative diagnostic evaluations for pathologies in the maxillary sinus are low-dose cone-beam CT and dental magnetic resonance imaging (MRI) using the black-bone MRI sequence [33,34]. Because anatomic variations that can cause stenosis of maxillary drainage pathways in addition to maxillary sinus pathologies should be evaluated, the imaging range should involve the natural ostium of the maxillary sinus.

ENT specialists do not recommend treatment for mucosal thickening less than one-third to one-half of the maxillary sinus height [20,21]. However, some dentists reported that mucosal thickening of more than 5 mm is associated with an increased risk of sinus outflow obstruction [35,36]. Carmeli et al. reported that mucosal thickening of <5 (11.1%), <10 (36.2%), and >10 mm (74.3%) was associated with the obstruction of the maxillary natural ostium (*p* < 0.0001) [35]. Shanbhag et al. found that the obstruction was associated with mucosal thickening of 2–5 (6.7%), 5–10 (24%), and >10 mm (35.3%, *p* < 0.001) [36]. Therefore, there are many cases in which the natural ostium is patent even when mucosal thickening is greater than 5 mm. We think that mucosal thickening greater than 5 mm does not matter when the natural ostium is patent because the ventilation and drainage of the maxillary sinus are performed through the natural ostium [30]. Dentists should pay attention to the status of the natural ostium. Patients with ECRS have a contraindication for maxillary implantation even if mucosal thickening is less than 5 mm. Therefore, we should judge whether patients are suitable for maxillary implantation according not only to mucosal thickening, but also to the patency of the natural ostium.

## 5. Conclusions

We showed that aberrant anatomical factors are predisposing risk factors for postoperative complications of the maxillary sinus. The paradigm for personalized medicine for patients with a maxillary edentulous jaw by ENT specialist and dentist cooperation is shifting from the assessment of maxillary sinus pathologies alone to including the assessment of anatomic variations, which can cause the impairment of maxillary sinus drainage in addition to maxillary sinus pathologies. Precise risk assessments are required, and further work is essential to verify whether the clinical outcome under this new proposal is superior to that under the traditional paradigm in order to provide more clinical relevance to this new proposal.
